# Macula Structure and Microvascular Changes in Recent Small Subcortical Infarct Patients

**DOI:** 10.3389/fneur.2020.615252

**Published:** 2021-01-07

**Authors:** Yungang Cao, Jueyue Yan, Zhenxiang Zhan, Yuanbo Liang, Zhao Han

**Affiliations:** ^1^Department of Neurology, The Second Affiliated Hospital and Yuying Children's Hospital of Wenzhou Medical University, Wenzhou, China; ^2^School of Medicine, First Affiliated Hospital, Zhejiang University, Hangzhou, China; ^3^Eye Hospital of Wenzhou Medical University, Wenzhou, China

**Keywords:** recent small subcortical infarcts, macular capillaries, retina, capillary densities, optical coherence tomography angiography

## Abstract

**Purpose:** This study aimed to assess the macula structure and capillaries in the macula and optic nerve head in recent small subcortical infarct (RSSI) patients.

**Methods:** This observational cross-sectional study included 40 RSSI patients and 46 healthy controls. Optical coherence tomography angiography was used to image the capillaries in the macula and optic nerve head. An inbuilt algorithm was used to measure the densities in the microvasculature of the macula [superficial retinal capillary plexus (SRCP) and deep retinal capillary plexus (DRCP)] and optic nerve head [radial peripapillary capillary (RPC)] and thickness around the optic nerve head, peripapillary retinal nerve fiber layer (pRNFL).

**Results:** Densities in RPC (*P* < 0.001), SRCP (*P* = 0.001), and DRCP (*P* = 0.003) were reduced in RSSI patients when compared with healthy controls. The pRNFL thickness was thinner (*P* < 0.001) in RSSI patients than healthy controls. In the RSSI group, the SRCP density significantly correlated with the DRCP density (rho = 0.381, *P* = 0.042). The pRNFL thickness displayed a significant relationship with the RPC density (rho = 0.482, *P* = 0.003) in the RSSI group.

**Conclusions:** RSSI patients showed interrupted capillary plexuses leading to its significant impairment and neurodegeneration. Our report provides insight into the macula capillary microcirculation changes in RSSI.

## Introduction

Recent small subcortical infarct (RSSI), a common radiological marker of cerebral small vessel disease (SVD), is one of the major cerebrovascular diseases in the aging community currently. RSSI, which affects the perforating arterioles, venules, and capillaries of the brain, causes cerebral vascular impairment (1, 2). With the clinical complications and unstable clinical symptoms associated with RSSI, foremost inhibition has been recommended to have the greatest influence on the general public and healthcare. Till now, there is still lacking reliable and reproducible imaging tools to monitor suppleness of the brain and cognitive function and to monitor asymptomatic participants who have a high probability of having ischemic stroke (3). Impairment of the microvasculature, as seen in lacunar infarction, has been implicated in the pathogenesis of ischemic stroke (4). Despite the improvement in the cerebral imaging modalities, the cerebral microcirculation still remains difficult to directly visualize the cerebral microcirculation, and postmortem findings are usually late-stage changes. Moreover, insufficient treatment options have driven the identification of structural biomarkers to help in the observation and prevention of participants in the very early phase of the disease.

The inner retina microvasculature provides a route to assess the cerebral microvasculature directly and non-invasively *in vivo*, because reports have shown that the retinal microvasculature shares similar physiological, embryological, and anatomical features with the cerebral microvasculature (5). Patients who have suffered from stroke have been reported to show a higher prevalence of retinal microangiopathic conditions when matched with healthy controls (6); ophthalmological findings such as retinal microaneurysms have been reported to be some of the clinical manifestations associated with ischemic stroke. Reports from the past decade used retinal imaging to study cerebral diseases such as pre-asymptomatic stroke (7–9). These reports showed that the retinal vasculature caliber, tortuosity, and fractal dimension reflect the cerebral vasculature during the disease cascade of ischemic stroke. Previous retinal vascular reports on ischemic stroke used fluorescein angiography (FA) to image and visualize the retinal vasculature. Due to the resolution of FA, imaging is limited to the superficial portion of the macula. Furthermore, it has been reported that retinal vascular changes usually seen on the fundus camera are late indicators of cerebral diseases such as stroke (10, 11).

Optical coherence tomography angiography (OCTA) is an imaging tool that provides an in-depth non-invasive imaging of the retinal microvasculature; it allows a three-dimensional view of the retinal microvasculature in different layers of the retina without dye injection (12); it also provides perfusion of the retinal blood flow. Additionally, the OCTA provides imaging and perfusion of the blood flow in and around the optic nerve head (ONH), which has been reported to serve as additional diagnostic parameters in systemic disorders such as neuromyelitis optica spectrum disorders (13).

Our study utilized the OCTA to measure and summarize the macula microvasculature in patients with RSSI; we also evaluated the macula thickness and microvasculature around the ONH.

## Methods

### Study Design and Participants

This observational cross-sectional study was conducted from November 2019 to June 2020 at the Second Affiliated Hospital and Yuying Children's Hospital of Wenzhou Medical University in China. The study was permitted by the Ethics Committee of Second Affiliated Hospital and Yuying Children's Hospital and adhered to the Declaration of Helsinki. All participants in our study brought written informed consent before being enrolled in our study.

The participants in this study were recruited from the Yuying Children's Hospital of Wenzhou Medical University from November 2019 to June 2020. RSSI was defined as a classic lacunar syndrome as previously reported (14). Cerebral imaging was also done to confirm the diagnosis and kind of stroke enrolled in our study (15). Where the clinical classification differed from the radiologic classification, cerebral imaging classification was used because the use of only clinical criteria may result in misclassification. Patients included in our study were in the age range of 35 to <80 years. Exclusion criteria also included: (1) patients with contraindications to MRI or unable to cooperate with ophthalmology examination; (2) except for atherosclerosis and small vascular disease, other causes such as cardiogenicity and vasculitis or patients with unknown causes of cerebral infarction; (3) patients with obvious medical diseases such as liver failure, kidney failure, heart failure, serious infections, and malignant diseases; and (4) patients with eye diseases (such as glaucoma, age-related macular degeneration, and diabetic retinopathy) or eye surgery (such as cataract extraction and laser surgery) that affects the retinal microcirculation.

Healthy controls, also finished with brain MRI and with a similar age range from the working staff of both hospitals, were enrolled in our study after consenting. The criteria for inclusion in the control group were as follows: absence of neurological disorders upon evaluation, an Mini-Mental State Examination (MMSE) score of ≥ 26 (cognitively normal), absence of glaucoma or any other eye disease, normal appearance of the ONH and normal thickness of peripapillary retinal nerve fiber layer (pRNFL), intraocular pressure (IOP) below 21.0 mm Hg, and no losses characteristic for glaucoma in visual field testing. Cerebral imaging was also done to rule out any visible cerebral disorders. Other exclusion criteria were as follows: (1) contraindications of MRI examination or inability to cooperate with eye examination; (2) head MRI showed stroke or white matter lesions (Fazekas score ≥ 2); (3) complicated liver failure, kidney failure, heart failure, severe infection, malignant disease, etc.; (4) patients with severe medical diseases; (5) patients with eye diseases (glaucoma, age-related macular degeneration, diabetes with retinopathy, etc.) or patients undergoing eye surgery (such as cataract extraction and laser surgery) that affects the retinal microcirculation.

### Data Collection for Clinical Parameters

Participants' basic and clinical information was recorded at admission, such as sex, age, stroke severity [National Institutes of Health Stroke Scale (NIHSS)], and body mass index [calculated as measured weight (kg) divided by the square of measured height (m^2^)]. Vascular risk factors comprised history of diabetes mellitus, hypertension, transient ischemic attack (TIA), coronary heart disease, cigarette smoking, and alcohol consumption. Ophthalmology-related indicators included IOP, visual acuity (VA), and fundus examination.

### Patient Assessment

Each patient enrolled in our current study underwent assessment of cognitive function using the MMSE by a well-trained neurologist.

The RSSI patients were patients with recent (within 3–4 months) clinical lacunar ischemic stroke. All patients were assessed by a well-trained neurologist (Dr. Han, a stroke physician). Recruitment, testing, and imaging of ischemic patients followed a previous report (14). Assessment of the severity of stroke was under the NIHSS (16); the classification of stroke followed the Oxfordshire Community Stroke Project classification (15).

### Measurement of Visual Acuity

Measurement of each participant's VA was done using the Snellen chart at a 3.2 m distance. Each eye (both right and left) was measured for its VA.

### Fundus Imaging

Fundus camera (CR-DGi; Canon USA Inc., Lakes Success, NY) was used to image the fundus and optic disc of all participants. Patients who presented with the following were excluded: retinal hemorrhages, exudates (soft or hard), macular edema, and swelling of the optic disc.

### Spectral Domain Optical Coherence Tomography and Optical Coherence Tomography Angiography Imaging

The imaging of the pRNFL was done with the spectral domain OCT (SD-OCT). OCTA was used to image the capillaries in the macula [superficial retinal capillary plexus (SRCP) and deep retinal capillary plexus (DRCP)] and ONH [radial peripapillary capillaries (RPCs)] The Avanti RTVue-XR tool (Optovue, Fremont, California, USA; software V.2017.100.0.1) was used for imaging. An inbuilt software in the OCT was used to measure the thickness, microvascular density, and area of foveal avascular zone (FAZ). For the inclusion criteria in our present study, high-quality images with signal quality (SQ) ≥ 6 were accepted according to the OSCAR-IB criteria (17).

### Statistical Analyses

Both eyes of each patient were included in the data analyses. Among the two groups, generalized estimating equation (GEE) was used to compare the OCT and OCTA parameters while adjusting for risk factors (sex, age, intereye dependencies, hypertension, and diabetes) and SQ. Association between OCT parameters was assessed by Pearson's correlation. *P* < 0.05 were considered as significant.

## Results

Seventy-five eyes from 38 RSSI patients and 92 eyes from 46 healthy controls were included for data analyses. One eye of RSSI patient was not included because it could meet the imaging signal criteria of our study (SQ < 6). A significant difference was seen in the best corrected VA (BCVA) when both groups were compared as seen in Table 1.

**Table 1:** Demographics and clinical information of RSSI patients and healthy controls.

**Parameters**	**RSSI**	**Healthy controls**	***P*-value**
Number	38	46	
Number of eyes	75	92	
Age, years	64.37 (8.56)	62.76 (6.46)	0.726
Gender (M:F)	26:12	31:15	0.921
Median NIHSS	1	–	<0.001
BMI (SD)	23.82 (3.12)	24.09 (2.91)	0.887
IOP (SD)	12.12 (2.41)	11.51 (3.90)	0.083
BCVA, LogMAR	0.058 (0.08)	−0.18 (0.02)	<0.001
**Medical history, no (%)**		
Diabetes	12 (31.5)	2	<0.001
Hypertension	15 (39.5)	5	<0.001
TIA	1 (2.6)	0	<0.001
Ischemic heart disease	2 (5.2)	0	<0.001

*NIHSS, NIH Stroke Scale; BMI, body mass index; IOP, intraocular pressure; BCVA, best-corrected visual acuity; SD, standard deviation*.

**Figure 1:**
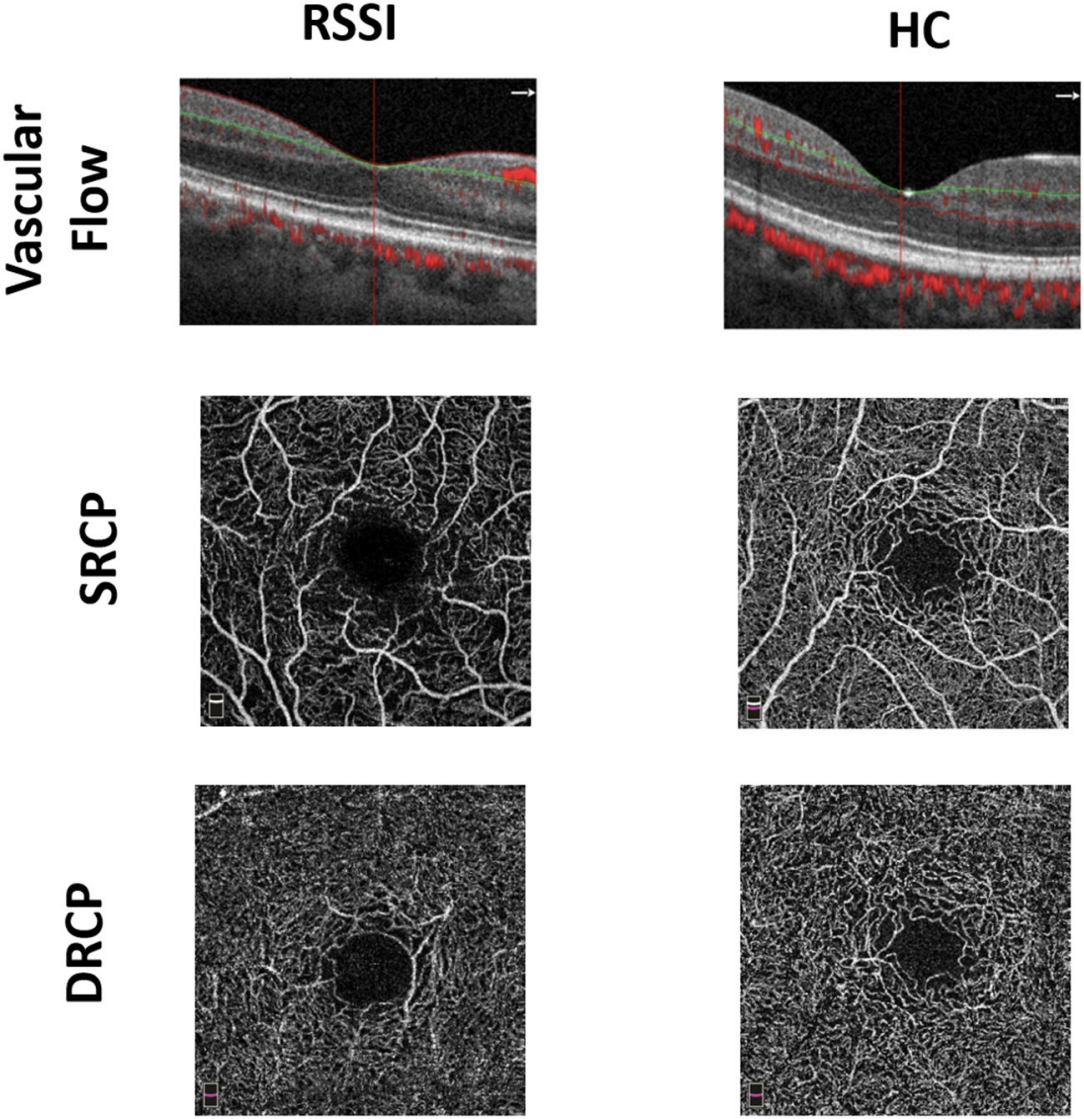
Representative en-face optical coherence tomography (OCT) angiogram of recent small subcortical infarct (RSSI) patients and healthy controls (HCs). The first row shows the vascular flow in the macula between the two groups. The second and third rows show superficial retinal capillary plexus (SRCP) and deep retinal capillary plexus (DRCP) plexus of RSSI and HCs, respectively.

Cross-sectional images from the macula OCTA with noticeable vascular flow motions through the angiograms of the macula were not the same among the two groups. RSSI patients showed reduced visible flow signals than did the healthy controls as seen in Figure 1. *En-face* angiograms between the two groups showed that the macular microvasculature in the RSSI patients was more interrupted in the SRCP than that in the healthy controls.

### Comparison of the Macula Microvasculature Between Lacunar Ischemic Patients and Healthy Controls

The SQ of the ONH and macula images was significantly lower (*P* < 0.001, Table 2) in RSSI patients when compared with the healthy controls.

**Table 2:** Comparison of the macular microvascular densities between RSSI patients and healthy controls.

	**RSSI**	**Controls**	***P*-value**
pRNFL, μm	109.19 (10.76)	116.79 (7.03)	<0.001
RPC density, whole (%)	48.86 (2.53)	50.48 (1.93)	0.001
SRCP, whole (%)	44.97 (3.60)	47.25 (1.38)	0.003
DRCP, whole (%)	48.82 (3.79)	52.08 (2.02)	0.002
FAZ (mm^2^)	0.29 (0.16)	0.22 (0.06)	0.025
Macula SQ	6.53 (0.82)	8.21 (1.01)	<0.001
ONH SQ	6.12 (0.51)	8.45 (0.97)	<0.001

*Data were adjusted for age, gender, hypertension, diabetes, intereye dependencies and signal quality (SQ). Data were shown as mean (standard deviation)*.

Densities in the RPC (*P* = 0.001, Table 2), SRCP (*P* = 0.003, Table 2), and DRCP (*P* = 0.002, Table 2) were significantly reduced in RSSI patients when compared with healthy controls. In the RSSI group, the SRCP significantly correlated with the DRCP (rho = 0.398, *P* = 0.015). The FAZ was significantly larger (*P* = 0.025) in RSSI patients when compared with the healthy controls.

The pRNFL thickness was thinner (*P* < 0.001, Table 2) in RSSI patients when compared with healthy controls. The pRNFL thickness displayed a significant correlation with the RPC density (rho = 0.401, *P* = 0.014) in the RSSI group.

### Association Between Macula Parameters and Clinical Variables

pRNFL thickness in RSSI patients inversely correlated with their VA (rho = −0.313, *P* = 0.039).

## Discussion

Reports have suggested the eye as a route to the brain (18, 19); moreover, retinal microvasculature has been reported to be associated with the cerebral microcirculation because both tissues share similar physiological, embryological, and anatomical features (5, 20). Our report utilized the OCTA to assess the microvasculature of the macula in RSSI patients and found that the macular vascular density is significantly reduced in the RPC, SRCP, and DRCP. Additionally, we showed that RSSI patients have significantly reduced pRNFL thickness and have a larger FAZ area than the healthy controls.

Previous reports used fundus photography to evaluate the retinal vasculature in ischemic stroke (9, 14). Nonetheless, using these imaging modalities limits the resolution and depth of imaging; moreover, these imaging modalities cannot visualize the deeper retinal layers in the retina and cannot give additional information on the capillary structure in the retina. OCTA is an imaging tool that offers a detailed non-invasive image of the retinal microvasculature; it allows a three-dimensional view of the retinal microvasculature in different layers of the retina without dye injection; it also provides perfusion of the retinal blood flow. Additionally, the OCTA provides imaging and perfusion of the blood flow in and around the ONH, which has been reported to serve as additional diagnostic parameters in systemic disorders such as neuromyelitis optica spectrum disorders.

Our current report showed that RSSI patients have significantly reduced SRCP density than healthy controls. Previous reports (7, 9, 21) used different algorithms to evaluate the photography in patients with ischemic stroke; these reports showed that patients with ischemic stroke have significant arteriolar and venular changes than healthy controls. Sprodhuber et al. (9) also showed that patients with ischemic stroke have significantly reduced retinal vascular density than healthy controls. The SRCP is within the retinal nerve fiber layer (RNFL) and superficial portion of the ganglion cell complex (GCC), which consists of both large vessels and microvessels (22) as seen via the fundus photography. Thus, reduced SRCP density in RSSI patients in our current study is congruent with previous studies, echoing the importance of retinal imaging in ischemic stroke.

A novel finding in our current report is the reduced microvascular density in the DRCP of RSSI patients when compared with healthy controls. Reports on OCTA studies have suggested that the DRCP makes up the end of the macula capillary plexus in which blood flows from the superficial capillary layers (retinal peripapillary capillaries and SRCP) and flows into the deep venules through the DRCP (22–24). Interestingly, our report showed that the SRCP density is significantly associated with the DRCP density; thus, reduced microvascular density in the DRCP of RSSI patients found in our current study may be due to the injury caused in the SRCP, which leads to the damage in the DRCP. The DRCP is positioned at the border of the inner plexiform layer (IPL) and outer plexiform layer (OPL), an area in the macular where the level of oxygen is significantly lower. As such, hypoperfusion may be easily prone to this area and maybe another reason for the reduced density in the DRCP of RSSI patients. Furthermore, deep capillary plexus has been reported to consist of capillaries (23) (more capillaries than superficial capillary plexus), which are responsible for oxygen diffusion in the tissue (25). The microvasculature in the deep capillary plexus is thinner and has a small cross section, making it sensitive to any disease that affects the retina; as such, any injury or insult to the capillary plexus leads to the tissue receiving less oxygen. Additionally, pericytes have been reported to play a pivotal role in the pathogenesis of lacunar ischemic stroke. Reports have shown pericyte alterations in the retinal capillaries of ischemic patients (26, 27). The reduced microvascular density in the deep capillary plexus of RSSI patients could also be as result of the pericyte alteration during the disease cascade.

RPCs are situated around the ONH and constitute a unique vascular network within pRNFL. The RPCs are also associated with metabolism of the RNFL and ganglion cell layer (GCL) (28–30). Because of their thin capillary anastomoses, RPCs are suggested to be susceptible to changes that occur in the ONH and macula (31, 32). Previous reports showed reduction of the pRNFL thickness in ischemic stroke patients when compared with healthy controls (33, 34); another report also showed optic nerve atrophy in patients with minor stroke (6). Furthermore, a previous report used the fundus photography to characterize the density of vessels around the ONH in ischemic stroke patients and found significantly lower values of the vessel area than in healthy controls (9). The authors suggested that retinal imaging could serve as a quantitative marker for cerebrovascular events. To the best of our knowledge, this is the first study to evaluate the microvascular density around the ONH in RSSI patients using the OCTA. Our report showed that RSSI patients had significantly reduced pRNFL thickness and RPC density than the healthy controls; our data also showed a significant association between the pRNFL thickness and RPC density in RSSI patients. The optic nerve has been reported associated with the brain (35); furthermore, the optic nerve is a boundary between the brain and the retina; thus, changes in and around the optic nerve mirror the changes that occur in the brain and retina. Besides, neural activity has been reported to be correlated with local blood flow (36); thus, the changes in the microvascular perfusion could be a result of the neurodegeneration or vice versa. We suggest that microvascular changes around the optic nerve mirror the neural and microcirculation in the brain of these patients. Longitudinal studies are needed to validate our hypothesis.

With the interaction between the neural activity and microvasculature, we suggest that the microvascular changes seen in our report may be due to the neurodegeneration associated with the disease cascade (37). Moreover, it has been reported that retinal vascular changes precede apparent cerebral changes seen on neuroimaging (38). With the connection between the activity of neurons and the flow of blood (39, 40), changes in the thickness of the pRNFL could lead to a secondary decrease in the density of microvessels seen in our current study.

It has been reported that about 30% of all stroke patients suffer from visual impairment (41). Our report found a significant correlation between the decreased pRNFL thickness and VA in RSSI patients. Although it has been suggested that patients with stroke have reduced RNFL thickness than have healthy controls (33, 34), an association between the reduced thickness and visual function has not been reported yet. The pRNFL, which contains axons (42), has been reported to play an important role in vision because of its proximity to the optic nerve (cranial nerve II). The association between pRNFL thickness and VA suggests that insult to the pRNFL thickness have a significant effect on the VA of RSSI patients.

Some limitations were seen in our current study. Our participants were Chinese; thus, our data cannot be translated across other ethnicities. Secondly, because of the observational, cross-sectional plan of our study, our study did not investigate the causative mechanism involved in our results. The OCT-A imaging technique requires that patients focus and work together with the examiner; therein, some of the images obtained were unsuitable for imaging because some patients could not focus and cooperate. Another limitation in our study is not evaluating other significant layers of the macula, which are associated with the neurodegeneration associated with the disease cascade such as the GCC. Nonetheless, our current study used the OCTA with an inbuilt software to assess the macula structure, which limited our macula structure thickness to the retinal sublayer thickness (RNFL) only. Reports with a segmentation algorithm of the retina are needed to elucidate the association between the macular thickness and microvascular densities. Another limitation is that our current study did not assess the association among the MRI parameters, OCTA measures, and clinical variables.

In conclusion, RSSI patients showed interrupted capillary plexuses leading to its significant impairment and neurodegeneration. Our report sheds light on the role of macula microvasculature and shows the global changes in macula microvasculature in RSSI. We also suggest that the OCTA could be useful to evaluate and monitor the microvasculature of the macula in patients with RSSI; nonetheless, longitudinal studies are needed to validate our report before the OCTA could be implemented as a screening tool.

## Data Availability Statement

The original contributions presented in the study are included in the article/supplementary materials, further inquiries can be directed to the corresponding author/s.

## Ethics Statement

The study was approved by the Ethics committee of Second Affiliated Hospital and Yuying Childrened Hospital and followed the Declaration of Helsinki. The patients/participants provided their written informed consent to participate in this study. Written informed consent was obtained from the individual(s) for the publication of any potentially identifiable images or data included in this article.

## Author Contributions

YC, JY, YL, and ZH: main authors and assisted with distribution as well as data collation and analysis. YC and JY: formal analysis. YC and ZZ: data curation. YC: writing—original draft. ZH: writing—review and editing. YL: supervision. All authors: contributed to the article and approved the submitted version.

## Conflict of Interest

The authors declare that the research was conducted in the absence of any commercial or financial relationships that could be construed as a potential conflict of interest.

## Abbreviations

pRNFL: peripapillary retinal nerve fiber layer
RPC: radial peripapillary capillary
SRCP: superficial retinal capillary plexus
DRCP: deep retinal capillary plexus
FAZ: foveal avascular zone
RSSI: recent small subcortical infarct
OCTA: optical coherence tomography angiography
SD-OCT: spectral domain optical coherence tomography
SQ: signal quality
IOP: intraocular pressure.
